# Response to Kestens *et al*. Comments on Melis *et al*. The Effects of the Urban Built Environment on Mental Health: A Cohort Study in a Large Northern Italian City. *Int. J. Environ. Res. Public Health*, 2015, *12*, 14898–14915

**DOI:** 10.3390/ijerph13030251

**Published:** 2016-02-24

**Authors:** Giulia Melis, Elena Gelormino, Giulia Marra, Elisa Ferracin, Giuseppe Costa

**Affiliations:** 1 Environmental Heritage and Urban Redevelopment Unit, SiTI—Higher Institute on Territorial Systems for Innovation, via Boggio 61, 10138 Torino, Italy; giulia.marra@polito.it; 2 ASL Torino 5, Local Public Health Agency, Piazza S. Pellico 1, 10023 Chieri, Italy; gelormino.elena@aslto5.piemonte.it; 3 SEPI Grugliasco, Epidemiology Service for ASL Torino 3, Local Public Health Agency, Via Sabaudia 164, 10095 Grugliasco, Italy; elisa.ferracin@epi.piemonte.it (E.F.); giuseppe.costa@epi.piemonte.it (G.C.)

The commentary from Kestens *et al.* [1] raises interesting issues about measuring contextual exposures and encourages new studies to incorporate them in their design: as a group of researchers, we strongly support their view and think that those useful reflections should be used as guidelines for future research.

We understand that our study [2] is limited in measuring exposure. We used, in fact, only administrative data, because they provided a unique opportunity for identifying the most promising urban characteristics able to affect mental health in the whole metropolitan population of Turin: this choice has been taken being aware that a deeper understanding of those influencing factors will be needed with future research to understand underlying mechanisms. According to what suggested by Kestens *et al.*, most of the recommendations presented in the Commentary to our paper have now been placed in our agenda, the main challenge will be how both administrative data on residential and health careers and data of individual daily mobility provided with the use of Information and Communications Technology (ICT) (including aggregate data on detailed characterization of exposure provided by sample based survey) can be integrated at the individual level. Apart from funding, additional problems come from the protection of privacy issue and from the need of renovated commitment from policy makers at the city level for such large-scale studies on the overall Turin population. 

Though being extremely cautious when inferring any causal relationship, we think that our main findings on the association between accessibility and better mental health should not be strongly biased by the limitation in measuring exposure to environments different from the residential one among more mobile people. When controlled for the number of years at same address, as a proxy for residential stability, and for sociodemographic variables, as proxies of lifestyles in main daily destination and residential trajectories, the estimated impact of accessibility on mental health could be at the most underestimated, deserving still attention for further investigation and policy making. For instance, it is true that workers may experience protective effects of the built environment in areas other than their residence, but we could still say that those effects go in the same direction as those who live in an inaccessible and poorly-served neighborhood. If you live in an inaccessible neighborhood with poor services, it is true that you may suffer less from this if you have to commute for work to a better served neighborhood, as this gives you the opportunity to satisfy your needs there; while you will have deeper negative effects if you have to travel long to reach the services every time you need it. Anyway, in the end the two effects go in the same direction when considering mental health, as in both cases you are forced to move from your neighborhood in order to gain access to basic facilities. When talking about the protective effects—the only ones we speculate about in the paper—derived from transit and density, we think there is no bias unless an underestimation of intensity, because thanks to the stratification for age and gender (that groups people with more or less chances to commute for work), we are able to highlight effects on the most disadvantaged groups, even considering that also commuters pay a price for their time spent in trips. In addition, controlling for socio-economic characteristics excludes bias for social position or employment.

We recognize that exposure to the residential neighborhood was not measured accurately, relying on a proxy which does not provide any details about where one is spending his/her daily time; therefore, we might have underestimated the positive effects of green areas and parks, of services and facilities, by ignoring the additional accessibility to them which is gained when you spend your time in a better neighborhood than your residential one.

Finally, knowing that age and gender influence both daily mobility and antidepressant consumption we decided to stratify analyses for gender and age not allowing to test interaction of those factors on the relationship between built environment and mental health. On the side of health inequalities, the association of accessibility with mental health remained the same when estimates were adjusted or not by individual sociodemographic characteristics; this means that accessibility was equally distributed by social position, at least in Turin. Moreover, no interaction on the mental health effect was found between employment status and accessibility by public transport, the individual and neighborhood variables with the most significant impact on antidepressant prescription after running the model; this means that mental health of the unemployed was not more vulnerable to low accessibility compared to employed. Both results support the conclusion that accessibility is not an issue for health inequalities, at least in Turin.
